# Challenges to the right to health in sub-Saharan Africa: reflections on inequities in access to dialysis for patients with end-stage kidney failure

**DOI:** 10.1186/s12939-022-01715-3

**Published:** 2022-09-05

**Authors:** James Tataw Ashu, Jackline Mwangi, Supriya Subramani, Daniel Kaseje, Gloria Ashuntantang, Valerie A. Luyckx

**Affiliations:** 1 Internal Medicine and Nephrology, Jura Bernois Hospital, Berne, Moutier Switzerland; 2 Nephrology and Hypertension Service, Geneva University Hospitals, Geneva, Switzerland; 3 Department of Law Science and Technology at the School of Law, Jomo Kenyatta University of Agriculture and Technology, Nairobi, Kenya; 4 Institute of Biomedical Ethics and History of Medicine, University of Zurich, Zurich, Switzerland; 5 Great Lakes University of Kisumu, Kisumu, Kenya; 6 Yaounde General Hospital Faculty of Medicine and Biomedical Sciences, University of Yaoundé I, Yaounde, Cameroon; 7 Faculty of Health Sciences, The University of Bamenda, Bamenda, Cameroon; 8 Department of Nephrology, University Children’s Hospital, Zurich, Switzerland; 9 Department of Paediatrics and Child Health, University of Cape Town, Cape Town, South Africa; 10 Brigham and Women’s Hospital, Harvard Medical School, Boston, MA USA

**Keywords:** Equity, Right to health, Human rights, Kidney disease, Priority setting, Rationing, Sub-Saharan Africa

## Abstract

Realization of the individual’s right to health in settings such as sub-Saharan Africa, where health care adequate resources are lacking, is challenging. This paper demonstrates this challenge by illustrating the example of dialysis, which is an expensive but life-saving treatment for people with kidney failure. Dialysis resources, if available in sub-Saharan Africa, are generally limited but in high demand, and clinicians at the bedside are faced with deciding who lives and who dies. When resource limitations exist, transparent and objective priority setting regarding access to such expensive care is required to improve equity across all health needs in a population. This process however, which weighs individual and population health needs, denies some the right to health by limiting access to health care.

This paper unpacks what it means to recognize the right to health in sub-Saharan Africa, acknowledging the current resource availability and scarcity, and the larger socio-economic context. We argue, the first order of the right to health, which should always be realized, includes protection of health, i.e. prevention of disease through public health and health-in-all policy approaches. The second order right to health care would include provision of universal health coverage to all, such that risk factors and diseases can be effectively and equitably detected and treated early, to prevent disease progression or development of complications, and ultimately reduce the demand for expensive care. The third order right to health care would include equitable access to expensive care. In this paper, we argue that recognition of the inequities in realization of the right to health between individuals with “expensive” needs versus those with more affordable needs, countries must determine if, how, and when they will begin to provide such expensive care, so as to minimize these inequities as rapidly as possible. Such a process requires good governance, multi-stakeholder engagement, transparency, communication and a commitment to progress. We conclude the paper by emphasizing that striving towards the progressive realization of the right to health for all people living in SSA is key to achieving equity in access to quality health care and equitable opportunities for each individual to maximize their own state of health.

**Supplementary Information:**

The online version contains supplementary material available at 10.1186/s12939-022-01715-3.

## Introduction

*“I don’t want to be seen denying any citizen access to dialysis” -* these words were spoken by a minister of health of a low-income country in sub-Saharan Africa when approached by clinicians for guidance as to how to allocate very few dialysis slots at the main teaching hospital in the country. Although the spirit of this statement in terms of emphasizing equitable access to health care should be applauded, true advancement of the human right to health for all is dependent the realities of implementation [1].

Daily challenges in access to high-cost care for common conditions such as kidney disease when resources are scarce acutely highlight the potential conflict between the rights of the individual and the rights of the broader community when resource allocation decisions must be made. Equitable access to expensive care often remains an aspiration, and the frequent lack of official guidance from policy makers regarding access to such care when it exists simply transfers decision-making dilemmas to the clinicians and families at the bedside. Similar tensions have arisen, described repeatedly in the media in recent months, as the world is grappling with the COVID-19 pandemic and how to equitably and fairly allocate limited hospital resources even in high income settings. Official triage guidelines have been developed in many countries which sanction restricting the right to treatment from some patients in favor of others, based most often on anticipated prognosis, and thereby depriving some individuals of the right to intensive care treatment [2]. In addition, the increasing recognition that the social determinants of health underlie critical vulnerabilities to SARS-CoV-2 infection and severity of COVID-19 is forcing long overdue reckoning about systemic inequity and discrimination in high income settings and the meaning of the right to health for all [3].

A broader discussion on the appropriateness of use of the human rights framework as applied to health is elaborated elsewhere [4, 5]. To tackle the question of the right to health systematically and more simplistically from the clinical perspective, in this paper we describe the example of access to dialysis, a life-saving but expensive treatment for end-stage kidney failure (ESKF), which illustrates the inherent practical challenges in realizing the right to health - interpreted here as the right to health care, and to stay healthy - in sub-Saharan Africa (SSA), where many health needs remain incompletely or inequitably addressed or unmet. We begin by describing the burden of kidney disease in SSA and discuss whether the high burden of ESKF may be due in some part to the omission of health systems to prevent kidney disease and to provide access to early screening and treatment, all of which could reduce this burden. Concepts and commitments regarding the right to health in SSA are then introduced. Based on a specific country case example, we further discuss how a human rights perspective may apply to the right to dialysis for patients with kidney disease, within the context of universal health coverage (UHC) and the sustainable development goals (SDGs), and the roles and responsibilities of the various stakeholders involved. We end with considerations and recommendations as a potential foundation for ongoing debate to inform future policy development.

### Main text

This narrative review originates with a clinical example where the right to health is invoked with the best intentions, but where the capacity to deliver on this right is limited (Table 1). Given that this example is not uncommon in SSA, to begin to grapple with this reality, the concepts of the right to health as it applies to kidney disease and kidney care in SSA is examined. Purposive literature review (search strategy outlined in Supplementary Data) and compilation of data from the Institute of Health Metrics [6] and the Global Kidney Health Atlas [7] was conducted to substantiate facts on the burden of kidney disease and highlight the relative neglect of kidney disease as a public health problem and the stark inequities in access to diagnosis and care globally. Relevant international documents describing the human right to health are reviewed. Subsequent arguments are then systematically built upon identification of relevant stakeholders and how the concept of the progressive realization of the right to health may apply to kidney care in SSA. Comments and feedback are incorporated from the perspectives of clinicians, social scientists, philosophers, public health experts and human rights lawyers attending an international workshop entitled “African Perspectives on the Human Right to Health”.

**Table 1:** The minister of health’s dilemma

*Dialysis had begun over a decade previously when a senior politician had required it prior to receiving a kidney transplant. Dialysis services continued limping along in the capital city, run by a very committed and knowledgeable nurse. She had since diligently kept a large ledger of patients who started dialysis, with the most common entry next to their names being “rest in peace” a short time after they began. Many patients likely died of infections or being unable to attend regularly given high out of pocket costs of transport to and from dialysis. Some patients managed to survive and became strong advocates for kidney disease. One in particular, who had to commute 250 km for dialysis to the single unit in the capital city from his home town, had been heard by the minister and a plan was developed to open a dialysis unit at the hospital in the town where this man lived.* *A small building was built and a dialysis water system paid for and installed. Five dialysis machines which had been donated years prior, somewhat rusty and with instructions written in foreign language that no one understood, were brought out of their boxes. Large volumes of disposable dialysis supplies were procured. The unit was to be run by a physician who had been sponsored to train in nephrology outside of the country, but at the last minute he took an opportunity to emigrate elsewhere, so a foreign nephrologist was asked to help with the start of the service. Because the water system, although installed by a “reputable” company from a neighboring country, was grossly inadequate, the system was highly contaminated and dialysis had to be delayed for over a year until the pipes were all replaced at extra expense. The initial patient advocate died shortly before dialysis began in his home town, from complications related to treatment delays resulting from his long dialysis commute.* *Soon it became apparent that the old donated machines were useless as they were breaking down constantly. The machines needed replacement, again at government expense and new supplies had to be purchased to replace the many that had expired unused because of the delays. Once the dialysis unit had safe water and functioning equipment a service developed, free at the point of care for all citizens. The service was to be shepherded by 3 foreign physicians until local nephrology capacity could be built.* *The projected cost per patient on long-term dialysis was over 150 times the* per capita *health expenditure in the country. Demand for the service rapidly escalated and soon choices were needing to be made daily at the bedside of desperate patients and families – which of several patients should get access to the limited capacity, based on which criteria, when they all technically had an equal right to treatment? Physicians and nurses struggled with the moral distress of having to shoulder the burden of these life and death decisions. At this point the minister was approached about development of guidelines governing access to dialysis and he responded by stating that every citizen had a right to dialysis.*	

### The burden of kidney disease in sub-Saharan Africa

Kidney disease could be considered a barometer for a country’s health. Risk factors for kidney disease extend across society and the life-course, from rich to poor, from industrialized to agricultural societies, from infections to non-communicable diseases [NCDs], from traditional to western medicine, from inherited to preventable conditions, and from reversible to irreversible disease [8]. The management of kidney disease extends from optimizing maternal health and fetal development, to public health prevention of infections and NCDs, to promotion of healthy lifestyle choices, to equitable access to quality primary care, and to highly expensive therapies including dialysis and transplantation (kidney replacement therapy, KRT) [9].

The burden of kidney disease is rising, given its’ associations with the social determinants of health, global inequities, occupational risks, worsening pollution and climate change [8, 10]. Globally it is estimated that around 10% of adults have some form of chronic kidney disease (CKD), a proportion that is likely higher in SSA [11]. Given the reliance on blood and urine testing for diagnosis of kidney disease however, and following the adage “what does not get measured does not count”, the global burden of kidney disease has been relatively neglected. The global focus has been trained on the “big five” NCDs considered to contribute to most NCD deaths collectively (cardiovascular disease and stroke, cancer, diabetes, respiratory disease and mental health) [8, 12, 13]. Global deaths attributable to kidney diseases have been estimated to range between 4 and 10 million [8]. CKD is now projected to become the 5th most common cause of years of life lost by 2040 [14]. The burden of acute kidney injury (AKI) is not known as it is harder to measure, estimated at 13 million cases and 1.7 million deaths per year in 2012 [15].

Globally over 3 million people with ESKF live on dialysis or with a kidney transplant [16]. The vast majority of these individuals (90%) live in high- or upper middle-income countries where most people who need these therapies can access them under UHC, and therefore incidence and prevalence rates tend to be relatively stable [16, 17]. It is anticipated, that the number of people needing dialysis will double between 2010 and 2030, with the greatest growth in demand from Asia and Africa [16]. In 2010 it was estimated that between 2 and 7 million people with ESKF died prematurely without dialysis or transplantation, predominantly in lower income settings [16]. Others [18] have extrapolated that in SSA only 1.5% of people with ESKF due to hypertension or diabetes (the most common risk factors) receive KRT.

Under 1% of the world’s dialysis population lives in SSA where 16% of the world’s population reside [19]. This disproportion highlights the lack of access to diagnosis and care for kidney disease rather than a low prevalence in SSA, given that rates of CKD are presumed to be higher than in other world regions [20]. The three modalities of KRT, namely hemodialysis (HD), peritoneal dialysis (PD), and kidney transplantation are available in SSA, although HD is the only modality available in most countries [7]. In a recent survey of nephrologists from 36 SSA countries, dialysis prevalence rates were found to range from zero in 2 countries (Central African Republic, Sierra Leone) to under 10 per million population (pmp) in 14 countries, and were over 150 pmp in only 6 countries (Gabon, Mauritania, Mauritius, Seychelles, South Africa, Sudan and Swaziland) [21]. Prevalence rates from North Africa ranged from 300 to 734 pmp and globally reach over 3000 pmp in Taiwan [22, 23].

Several factors are responsible for the low numbers of people receiving treatment for ESKF in SSA, including inadequacy of dialysis infrastructure, lack of reimbursement or government subsidies for most dialysis programs and severe shortage of trained nephrology personnel [7]. Transplantation is also rarely available in SSA for similar reasons. Extrapolating from the global average ESKF prevalence of around 500 per million population [23], and a total population in SSA of 1.033 billion (2016), around 516,000 people would need KRT. Although no SSA dialysis registry exists, the survey suggested that the actual number of people receiving dialysis is less than 10% of this number [21] implying many people die without treatment [24].

Annual global reimbursement for dialysis (excluding out-of-pocket payments) is around 57 billion US dollars, of which 80% is from high-income, 17% from middle-income, and 3% from low-income countries [17]. Where UHC for dialysis exists (either state funded or through mandatory insurance), treatment for ESKF consumes 2–4% of national healthcare budgets on average, with a population prevalence of around 0.15% [25]. This expenditure is disproportionate, but has been accepted by society and decision makers given that KRT is life-saving, and if not covered leads to financial hardship [26]. In lower income settings, however, the opportunity costs for the health system are extremely high^.^. The low proportion of government spending in low income countries reflects the frequent reliance on out-of-pocket payments for ESKF care, mostly in private centers [27–29].

A recent systematic review of outcomes in patients with ESKF in SSA reported that among newly diagnosed patients who began dialysis (incident patients), only 1% was known to be still alive at 1 year [30]. Many patients discontinued dialysis when they no longer could pay for treatment, likely leaving behind impoverished families. Indeed, kidney disease is a major cause of catastrophic health expenditure (CHE) globally [31]. In lower-middle income and low income countries, which reflects most of SSA, kidney disease was the leading (83.3 million people) and second most common cause (3.8 million people, after infections) of CHE respectively [31]. In low-income countries the number of individuals experiencing CHE induced by kidney disease was 10 times that for cardiovascular disease or diabetes, which are currently considered priority NCDs [32]. Access to care is therefore highly inequitable across SSA and kidney disease imposes significant financial hardship on individuals and families.

Recognizing this, some governments in SSA do provide some funding or subsidize ESKF care, but dialysis units tend to be concentrated in urban centers, are faced with infrastructural challenges and are insufficient to meet the clinical demand (examples illustrated in Table 2) [34–36]. In Sudan, dialysis is offered to all, however at a reduced frequency (dose) from 3 to 2 sessions per week in order to accommodate more people [37]. Despite access for all in Sudan, it is likely that some patients remain undiagnosed, and it is known that some decline treatment because of remaining financial and logistical barriers e.g. transportation, need to relocate etc. [37–39] Kenya has recently committed to provide dialysis in all counties, although the majority of dialysis is still provided by the private sector. [oral communication, Dr. A Twahir, AFRAN meeting 2019] In contrast, South Africa has adopted an official rationing strategy where only those patients with ESKF who are deemed stable enough to be successfully transplanted (around 30%) are eligible for state-funded dialysis [40]. This rationing approach was tested in the constitutional courts in 1997 when a 41 year old man with diabetes and heart disease (i.e. not transplantable) was not accepted for state-funded dialysis [41]. The final ruling concluded that despite the patient’s right to life, if the state were to provide dialysis for all it would be unable to fulfill its’ other constitutional health responsibilities, and therefore should not prioritize dialysis over other illnesses and prevention programs [41]. The court did however suggest that inherent to the ruling, there was an obligation on the state to progressively expand access to dialysis. The progress in the public sectors has been very limited over the past 2 decades [42].

**Table 2:** Examples of state approached to dialysis provision in sub-Saharan Africa

	Expected benefits	Potential harms	Impact on equity	Expected cost	Country example	Practical issues	Cautions	Burdens for patients
**Universal coverage of dialysis for AKI and ESKD**	Potential for all to benefit	Diversion of funds to kidney disease, away from other diseases	Good for patients with kidney failure Kidney disease prevention programs often not in place	Expensive	Sudan, Zanzibar, Malawi, Kenya	Reduce dialysis frequency to reduce costs and increase capacity Infrastructure limited, in effect dialysis may not be available to all	Reducing dialysis frequency may not be acceptable for all Donations may drive program	Patients may need to move for dialysis, lose jobs, etc. Transport (and medication) costs not covered
**Government subsidy for dialysis**	Potential for all to benefit	Diversion of funds to kidney disease away from other disease	Tends to favour those with resources to pay	Expensive	Cameroon, Senegal, Ethiopia	Periodic stock outs when companies decline to deliver supplies because of late payments	Dialysis twice a week Reliance on single supplier, corruption	Large out of pocket expenses remain ($10–20 per treatment, medication, transport)
**Universal coverage for AKI only**	Saves lives of patients with AKI	Diversion of funds to kidney disease away from other disease	ESKF patients not dialyzed	More cost effective than dialysis for ESKF, short term treatment, may not require much infrastructure	South Africa, Ethiopia	Dialysis not available everywhere	If AKI does not recover must withdraw dialysis	Out of pocket expenses
**State coverage under limited conditions**	Benefit for those eligible to receive treatment	Ineligible patients die	May exacerbate inequities especially for poor, vulnerable, sick	Prioritise peritoneal dialysis (cheaper)? Prioritise Transplant (most cost-effective)?	South Africa	Patient selection process may impose delays Need good palliative care services	Need clear transparent guidelines, community engagement, appeal possibilities	Anxiety, distress, fear, helplessness, lack of understanding
**No state coverage**	Limited to rich few	Inadequate informed consent will lead to catastrophic expenditure and death	Exacerbates inequity	Left to market forces	Nigeria, Democratic Republic of Congo, Burundi	Generally only in large cities, very expensive	Lack of government regulation frequent,	If not fully informed poor patients may not realize life-long expenditure required

Table adapted with permission from [72] and presented as poster at the International Conference Clinical Ethics & Consultation (ICCEC) Oxford, 2018

To the average person living in most SSA countries, KRT remains unaffordable due to lack of UHC. Where state support is available, dialysis and transplant rationing, either implicit (as illustrated in Table 1) or explicit (as in South Africa), remains a significant limitation.

### Contextualizing the right to health in sub-Saharan Africa

The right to health was first mentioned in the preamble of the Constitution of World Health Organization (WHO), adopted in 1946 and came into force in 1948. In Africa, all states are member states to the WHO and as a consequence are bound by the provisions of WHO Constitution [43].

In 1948, the right to health was once again recognized and guaranteed in the Universal Declaration of Human Rights (UDHR) under Article 25 [44] Although the UDHR applies to all people, in all countries, it is not legally binding. However, the protection of the rights and freedoms set out in the UNDHR have been included in many international covenants, regional covenants, national constitutions and domestic legal frameworks.

At the international level, one of the most authoritative provisions on the right to health is Article 12(1) of the International Covenant on Economic, Social and Cultural Rights (ICESCR) which provides *“The States Parties to the present Covenant recognize the right of everyone to the enjoyment of the highest attainable standard of physical and mental health”* [45]. In Africa, 51 out of the 55 member states to the African Union have ratified the ICESCR [46].

At the regional level, the right to health in Africa is recognized and guaranteed under Article 16 of the African Charter on Human and Peoples’ Rights (Banjul Charter) which states that 1) “*Every individual shall have the right to enjoy the best attainable state of physical and mental health”; and 2) “State Parties to the present Charter shall take the necessary measures to protect the health of their people and to ensure that they receive medical attention when they are sick”* [47]*.*

As per the African Union treaty ratification table, 54 out of 55 member states have ratified the Banjul Charter [48]. All governments in Africa therefore, through their ratification of the ICESCR or the Banjul, Charter, recognize health as a right.

**Fig. 1:**
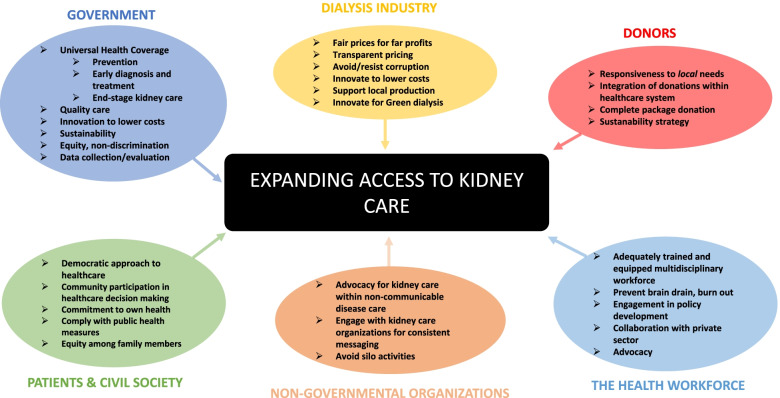
Role of stakeholders in expanding the right to kidney care

Recognition of the right to health implies that every member state is obliged to ensure access to timely, acceptable, and affordable health care of appropriate quality within the limitations of their resources, as well as to provide for the underlying determinants of health such as safe water, sanitation, food, housing, health-related information, and education, and gender equality [49]. The right to health contains the freedom to choose to receive treatment and the entitlement to protection of health, health information and access to quality treatment, without discrimination (Table 3) [49]. The right of the population to participate in health-related decisions is also included as an entitlement [49]. This right of participation should extend to all stakeholders in health care, to optimize realization of the right to health through practical planning, implementation, evaluation and iterative feedback to ensure progress (Supplementary Table 1, Fig. 1) [50].

**Table 3:** Core elements of the right to health*

Component	Dimensions	Relevance for dialysis
Availability	• Public healthcare facilities • Health care goods and equipment • Trained healthcare professionals	• Existence and location of public health facilities • Dialysis services available in public health facilities (renal unit) • Trained clinicians and nurses in delivering dialysis care
Accessibility	• No discrimination • Physical • Affordable • Information	• Equitable access for all, including children • Geographic location of dialysis centers • Reduce out-of-pocket expenditure, including ancillary costs e.g. medication, transport • Transparent communication about potential rationing
Acceptability	• Cultural • Gender • Religious	• Respect needs e.g. separate male and female areas in some countries
Quality	• Safe • Effective • Patient-centered • Timely • Equitable • Integrated • Efficient	• Infection control, building safety, respect curfews • Enough dialysis provided to keep patients safe especially if reduced frequency provided • e.g. adapt to patient’s work schedule if possible, Jehova’s witnesses • Avoid delays in emergencies (usually patients seeking funds, infrastructural failures) • no discrimination • Horizontal, embed dialysis within NCD programmes • Avoid waste, cost-awareness

*summarized from [33]

Human-rights based approaches have evolved to address inequalities and to progressively permit full participation of all individuals in social, economic and cultural aspects of life [51]. Specific vulnerable groups highlighted in United Nations and WHO documents on the right to health include women, children and adolescents, the elderly, people with disabilities, indigenous peoples, migrants and people living with HIV/AIDS, however many other vulnerable groups and individuals exist [49, 52].

It is clear world-wide that there are gaps between the recognition of the right to health and the practical realization of this right. These gaps are more evident in lower income settings, such as SSA. Most countries in SSA do have strong institutional health policies in place [53, 54], but a lot remains to be done towards the realization of the right to health in every sense. Importantly, the right to health does not mean that each individual has a right to perfect health, but each should have a right to their highest attainable state of standard of healthcare services available in the particular state [49].

### The challenge of lack of adequate resources in realizing the right to health

Although the right to health is recognized as a fundamental human right indispensable for the exercise of other human rights, the obligation on governments when it comes to its realization is not immediate but progressive [4]. Progressive realization recognizes that resources are required to comply with obligations that come with recognizing health as a human right, and not all governments can meet these obligations immediately because of financial constraints. Financial constraints are a reality for most countries in Africa: 24 countries in African are categorized as low income countries (LIC), 22 are categorized as lower middle income countries (LMIC), 7 countries are categorized as upper middle income countries (UMIC) and one, Seychelles, is categorized as a high income country (HIC) [55].

While progressive realization means that meeting the obligation is not immediate, it requires that the government must use the maximum of its available resources for the realization of the right to health. Furthermore it requires that governments have a specific and continuous obligation to move as expeditiously and effectively as possible towards the full realization of the right to health. With progressive realization there is no room for retrogression and where retrogression occurs, the government of the day has the burden of proving that such retrogressive measures have been introduced after the most careful considerations of all alternatives and that they are duly justified. There are however two exceptions to the general rule on progressive realization for countries that are State Parties to the ICESCR. Firstly, State Parties have an immediate obligation to ensure the right to access to health facilities, goods and services without discrimination of any kind [56]. Secondly, State Parties have an immediate obligation to take deliberate, concrete and targeted steps toward the full realization of the right to health [56].

Guaranteeing the right to health in SSA therefore comes with several challenges, one being lack of adequate resources. To appreciate how the challenge of lack of adequate resources undermines the realization of the right to health, we must we must contextualize it under the essential elements of the right to health, which include: availability, accessibility, acceptability and quality (Table 3) [52]. In SSA public health care facilities, goods and services are not available in sufficient quantity to meet the health needs of the population. In the context of dialysis this would be evidenced first by the lack of enough dialysis infrastructure to serve the number of people requiring it, and second even where there is dialysis infrastructure, there is a shortage of trained medical health professionals, water or electricity to deliver dialysis. Under these circumstances, physical accessibility and economic accessibility as essential elements to realization of the right to health are compromised. Quality and acceptability are challenging to achieve and maintain when infrastructure is insufficient, or when health care workers are lacking or inadequately trained or supported. These circumstances become even more difficult with respect to kidney disease, which is dependent on awareness, access to diagnosis, and consistent access to basic treatment or life-saving KRT. Practical barriers such as financial constraints, transparency and corruption in addition to lack of strategic planning often contribute to precluding the realization of the right to health.

#### Dialysis as an example of the tension between the right to health and equitable access to expensive health care

**Fig. 2:**
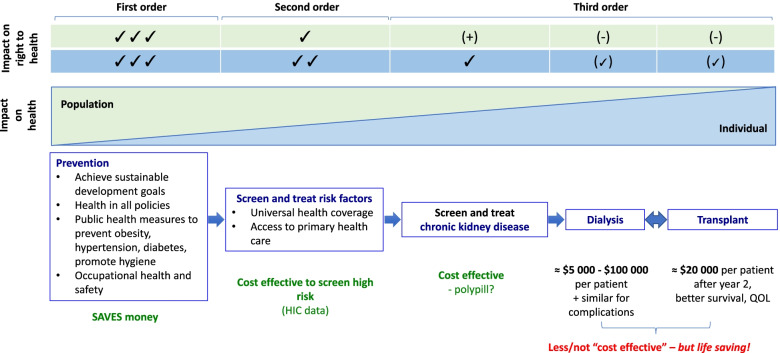
Spectrum of rights and obligations chronic kidney disease under resource limited conditions

Table 1 describes the development of dialysis services in a low-income country in Africa, where in theory every citizen has the right to dialysis, however the very limited infrastructure and lack of trained health care workers make the realization of this right impossible for most. The typical response when such country examples are discussed in HICs is that the LICs should not be providing dialysis to anyone [57]. LICs should focus on other priorities given the disproportionately high costs. This response implies that patients with expensive diseases have a lesser right to health care than those with less expensive diseases in lower income settings. It is true that the definition of the right to health (which necessitates prevention of disease as well as access to health care) includes the acknowledgment that the same rights may not be immediately achievable by all countries, but the definition also includes the expectation of progress towards more comprehensive inclusion of health conditions such that over time all citizens should have an equitable right to health care [50, 58]. From the individual’s perspective, this may mean that some individuals may have reduced realization of their right to (expensive) health care compared to others who may require more affordable care at a given time point. From the population perspective, upholding the right of an individual to expensive health care e.g. dialysis, however, may compromise the rights of many others to less expensive but equally life-saving health interventions (opportunity cost) e.g. vaccinations (Table 2, Fig. 2). Equity dilemmas also arise when NGOs and external organizations claim that dialysis is a « human right » and set up or support vertical programmes to deliver dialysis that are not horizontally integrated into the health system. These programmes do save some lives and therefore have intrinsic value, but exacerbate inequities in access to care, and risk destabilizing the local health system by diverting resources or generating expectations of « rights to care » that cannot be realized at scale.

Clinicians dealing with expensive care are at the interface between economics, individual health, and population health, as well as the many other sectors that affect health. The crucial aspect to be deliberated upon is whether the right to health is inalienable and must be met under all circumstances, or whether the human right to health becomes irrelevant under conditions of resource scarcity. Does the population have a greater right to being treated for tuberculosis or to be vaccinated or to have a road built or to go to school, or does the individual have a greater right to dialysis for the same money because it immediately saves a life? These conundrums arise also on a background of pervasive structural inequities which augment disease risk, and which become further exacerbated regardless of the choice made. Transparency and fair priority setting may be the most acceptable method for decision-makers to manage the competing rights of individuals and populations and to strategize how best to achieve equity across disease states [59, 60].

### Potential solutions towards enhancing the right to kidney care

From a clinical and public health perspective, the facts illustrate that realization of the right to dialysis for ESKF patients in SSA is far from a reality at present. A multipronged approach and good governance are required to address the inequities in access to kidney care across the spectrum of disease [61]. Reduction in demand for KRT through reducing the burden of ESKF is likely to be the most affordable strategy to reduce inequities in access to care between patients with kidney disease and those with other diseases, and within the spectrum of severity of kidney disease itself. Prevention of kidney disease is key and cannot be separated from the necessity for UHC. In addition, recognition of the roles and obligations of multiple stakeholders (Fig. 1, Supplementary Table 1) will facilitate accountability, transparency, participation and respect, all necessary to improve equity in access to prevention and care for kidney disease. These elements will be discussed individually in what follows.

#### The right to “stay healthy” – prevention of kidney disease

Access to KRT once kidneys fail requires a per capita health expenditure many times more than that allocated to other individuals, and therefore clearly full coverage of KRT for all who require it in SSA is currently not feasible (Table 2, Fig. 2) [57, 62]. However, given that ESKF may result, at least in part, because of prevailing social/structural factors and failure of the health system to protect kidney health, there must be some accountability on the part of the decision-makers with a commitment to reduce the overall burden of kidney disease [63]. Priority setting processes concluding that certain conditions are too expensive to treat, and global programmes calling for action on few priority diseases may risk diverting attention from many other « lesser » priority conditions, which simply on the basis of equity and solidarity still require attention. The right to health care should not be an « all or nothing » consideration, but instead, various orders of the right to health, aligned with current resource availability, could be considered in lower income settings, including many countries in SSA, as steps towards progressive full realization of the right to health (Fig. 2). The first order of the right to kidney health would include protection of health, i.e. prevention of risk of disease through public health and health-in-all policy approaches. The second order right to kidney health would include provision of UHC to all such that risk factors can be effectively and equitably detected and managed early to prevent kidney disease progression or development of complications. Access to expensive care such as dialysis and transplantation may need to be realized progressively as third order rights to health for people living with kidney disease.

Prevention of kidney disease is possible and cost-effective, and arguably the necessary public health interventions such as reduction in salt, sugar and fat intake, reduction in smoking, promotion of physical exercise, access to vaccination and other infectious disease prevention strategies should not be negotiable within the right to health in any country and should be realizable for every citizen [10]. Achievement of the SDGs is also a key component in realization of the right to health and reduction in poverty, improvement in nutrition, gender equity, access to quality education and dignified employment which would go some way to reducing kidney disease risk [8]. Major risk factors for kidney disease are known and case-finding by screening high-risk groups is cost effective [64, 65]. A large proportion of kidney disease is controllable with cheap generic medication in addition to adherence to healthy lifestyles, which should be deliverable under basic UHC and primary health care. Such strategies are key components of horizontal programmes to reduce the overall burden of NCDs and therefore should be integrated within the health system and will have benefits beyond kidney health. If the basic rights to prevention and early detection and treatment of kidney disease are realized, it is reasonable to expect that the incidence of ESKF will decline over time and the demand for expensive tertiary care should decline.

Programmes such as dialysis may not be affordable initially, but in the era of globalization and the availability of HD at least in the private sector in all SSA countries, this cannot be ignored. As programmes grow incrementally, necessary “gatekeeping” would require admission from governments that they cannot realize all rights to health and must introduce transparent priority setting [60]. Priority setting will de facto deprive some of the “rights to their best possible state of health” at any given time, and may even lead to death, while favoring others [66]. Transparent engagement with the community is necessary to ensure understanding and to communicate equity and justice implications of such strategies. Respect for patient dignity is fundamental to respecting the right to health and health care [67]. For patients with kidney disease this respect includes raising awareness of the risks of kidney disease, and the importance of self-care and adherence to healthy life-styles, ensuring equitable access to effective primary care, fully informing them of the implications of available treatment options, and provision of palliative care if active treatment is not possible. If the decision is taken to provide treatment free at the point of care, to fully realize the patient’s right to their highest achievable state of health, other costs necessitated by dialysis such as transportation, medication, catheters, surgeries must be included. Coverage for dialysis itself alone is not enough. Ancillary costs are frequently not covered however [37].

#### Universal health coverage and the right to health care for kidney disease in SSA

Priority setting has been emphasized as the fairest way to progressively achieve and expand UHC [57]. UHC in its truest sense is intrinsic to the realization of the right to health. The goals of UHC are 3-pronged and include 1) expansion of priority services; 2) inclusion of more people; 3) reduction of out-of-pocket payments [57]. At face value, provision of dialysis has been deemed an “unacceptable” trade-off in countries where UHC for basic health care needs has not yet been achieved, given the significant opportunity costs [68]. When one considers these 3 pillars in SSA, however, the lack of data about kidney disease makes decision making difficult. Whether kidney disease is indeed a priority is not known. The burden of NCDs is rising rapidly in SSA, risk factors for kidney disease are common. Many people are dying from lack of access to KRT, and a great many more have insufficient access to early diagnosis and treatment which could prevent progression to ESKF. In terms of financial risk protection, kidney disease is a leading case of CHE [31]. The 3 pillars of UHC are therefore highly relevant to kidney disease in SSA, but whether this is enough to justify inclusion of the entire spectrum of kidney disease therapies under UHC now must be determined in context by each country. Each country’s health authority will need to develop a financial model to ensure its sustainability and ensure fair and appropriate allocation of health care dollars [69]. Strategies such as inclusive participation in sustainable financing, where people pre-pay according to ability into a non-profit social insurance scheme, and the state pre-pays for indigents and subsidizes others as necessary safety-nets, may make UHC possible even in lower income settings. Quality and sustainability should also be fundamental components of UHC. In addition, it is important that in pursuit of the goal of national UHC, efforts must not be focused on quick wins and aggregate gains, but that true extension of good quality, accessible and affordable health care meets the needs of the most vulnerable populations such that human rights of all are upheld [70].

With dialysis and transplantation being highly unaffordable in most LICs and LMICs, achieving UHC with priority placed on early case finding through targeted screening of at-risk individuals, and early treatment of risk factors for kidney diseases progression, will enable timely access to care and prevent thousands of households from falling into poverty due to kidney disease [71].

#### The role of stakeholders in expanding the right to kidney care

##### Governments

There is very little regulation governing dialysis practice in most countries in SSA. As resources become available, governments in SSA must grapple with how and whether to begin to provide KRT [72]. Some countries in SSA, such as Kenya, have decided to include dialysis under UHC as it is rolled out over the coming years, based on a sharp rise in dialysis claims to the national health insurance fund [73, 74] The short, medium and long term implications of such a programme will require close monitoring and evaluation to quantify the benefits but also to identify any unintended harms, especially in terms of equity. Accurate and timely data collection will be required. Provision of KRT under UHC is being considered in other low-income settings. In India for example, in an endeavor to achieve UHC, free dialysis has been offered to those living below the poverty margin [75]. Ethically the question arises whether this level should be defined as an absolute number, or rather as that threshold beyond the purchasing power of an individual. Financing such programmes will require a transparent fiscal system, innovations to lower costs of HD without a compromise in quality, and better access to PD and/or transplantation which should cost less [76]. Proactive engagement with all stakeholders, including industry is necessary to provide affordable and scalable modalities of KRT such as PD. [77] From the patient perspective PD could be made available in remote locations, requires less dialysis time, provides better working flexibility and quality of life, provided barriers such as patient hygiene, the cost of consumables etc. are correctly addressed (for example local manufacturing of consumables) up front [78].

##### Dialysis industry

The dialysis industry is largely in the hands of monopolies and annually generates multiple billions of dollars in profit and continues to grow [79]. Pricing and sustainable delivery of dialysis supplies are major barriers to equitable access to dialysis in SSA. Given the enormity of the profit margins it is difficult to understand how prices cannot be lowered (through various strategies, including possible local manufacture of supplies) to permit improved access. Given that dialysis costs are largely paid for through public funds or taxation globally, the principle of solidarity demands that the dialysis industry has an obligation towards facilitating the right to dialysis everywhere. Governments need to be aware of and be proactive to curb corruption and profiteering from companies and middle-men given that by its life-saving nature, pricing of dialysis and the attendant supplies is vulnerable to manipulation. Corruption must also be measured and tackled at the individual level, as the expectation of payment of bribes by patients has been found to be an important barrier to care in SSA [80].

The COVID-19 pandemic has alerted the world to inequities in access to therapies and to vaccinations. Lower income countries are still waiting in line behind the richer countries as they buy up available vaccine stocks [81]. The COVAX initiative, however, is a global collaboration set up with the express purpose of addressing equitable access to COVID-19 vaccines. Such an initiative may seem more achievable for dissemination of a vaccine which requires 2–3 doses per person, than for dialysis which is required 2 to 3 times per week life-long. The scale of vaccines required for COVID-19 is however much larger than for ESKF, and therefore COVAX should serve as a precedent to tackle other global inequitably distributed therapies such as dialysis [82].

##### Donors

Over the past decade, health aid to SSA has risen more than other economic aid [83]. Although the motives of such donations or other activities driven by donors have been questioned by some, health gains have been achieved [83]. As illustrated in Table 1 however, donations of equipment may create a demand for a service such as dialysis, which can be problematic when not integrated within the health system [61]. At face value donations or donor-driven dialysis programmes appear to meet a clinical need that would otherwise go unmet, however multiple challenges arise with donations from HICs to LMICs which may impact the right to health care of the local population [84]. Donating part of a package (e.g. a machine) with no assurance the other parts are in place (e.g. dialysis disposables), or that technical expertise exists locally to maintain machines often leads to failure of the service. In addition, donors should have an obligation to integrate sustainability of the service once the donations (equipment, services) come to an end. Sudden collapse of a donor-driven service, after creating an expectation in the community, may be morally worse than never having set up the service in the first place as this creates great inequity and undermines trust in the broader health system.

#### The health workforce

The healthcare workforce is the cornerstone of any health care system. An adequately trained and sufficiently staffed workforce is essential to deliver on UHC. Implementation of policies to prevent, detect and treat kidney disease require appropriate resource allocation and a well informed and equipped health care workforce, who are the implementers of the patients’ right to health. As illustrated in Table 1, despite attempting to respect the rights of every citizen to receive dialysis when required, given the limited resources and infrastructure, clinicians were faced with having to take decisions as to which patient’s right to dialysis should be realized and which not. The need for such decision-making requires clear and consistent criteria for treatment eligibility which must be agreed upon by diverse stakeholders [40]. Development of such criteria, although supporting the concept of rationing of health care, at least promotes greater equity and may reduce moral distress. Health care workers should also be protected by the health system and have adequate resources to permit execution of their duties of care without being subject to unnecessary violence from patients and their families or moral distress [85]. SSA has a recognized shortage of health care workers of all cadres. There is an important lack of physicians, nurses and other health care professionals with expertise in managing kidney disease [86]. Some of this lack may be attributable to brain drain as a result of moral distress experienced daily by health care workers [85]. Transparency and engagement in policy development and decision making are key to empower health care workers to communicate and act, while permitting them space to advocate and to provide feedback. Engagement must also include health care workers in the private sector.

#### Patients and society

A component of the right to health is the right to participation. People in SSA have the highest rates of dissatisfaction with their health care and have the lowest perceived health status compared to people in other world regions [83]. A recent qualitative study conducted among women receiving maternity care in Tanzania revealed that women were aware that their right to health was not always respected [67]. This was illustrated by the expression of expecting to be “received well”, being treated equally and being well informed about their care. Patients and communities therefore have the obligation to make their needs known and to participate where possible in health care decision making, as well as to take steps to optimize their own health. This democratic approach to health care is not common in SSA but is fundamental to a human-rights based approach [87].

## Conclusion

Realizing the right to health and the equitable right to healthcare in low-income settings is not straightforward. The right to stay healthy (protection of health) is underscored by the SDGs which aim to achieve “healthy people living on a habitable planet” [88]. How to operationalize the right to health for patients with kidney disease in SSA is a challenge, but not insurmountable. In the absence of resources to provide equitable and sustainable KRT for all who require it, the obligation on the state to provide affordable disease prevention is a key component of respecting their right to achieve the highest possible state of health [61].

Transparent priority setting is required to improve equity across all health needs in a population, however this process, in which “rationing” is inherent, per se denies some the right to health. It is important that governments acknowledge that diseases may be too expensive to treat once they reach advanced stages, but that many are preventable or manageable at much lower cost. Entire disease groups therefore should not be marginalized in the priority setting process. The right to health care also implies the right to *quality* care, which is often not achieved [89]. Monitoring and evaluation processes must therefore be in place to ensure that quality care is deliverable and is delivered. Increased financial commitments on the part of governments in SSA are essential not only to fund prevention, case-finding, early detection and treatment of kidney disease, but also to progressively improve patient access to safe and affordable dialysis, to gather local epidemiological data on kidney disease causes, determine the true incidence and prevalence, and to train and retain nephrology personnel in the region. All stakeholders should exercise their rights to public participation and hold governments accountable for their decisions and to their commitment to progress. Striving towards the progressive realization of the right to health for all people living in SSA is key to achieving equity in access to quality health care and equitable opportunities for each individual to maximize their own state of health.

## Supplementary Information

**Additional file 1.**

## Data Availability

not applicable.

## Abbreviations

AKI: Acute kidney injury
CHE: Catastrophic health expenditure
CKD: Chronic kidney disease
ESKF: End-stage kidney failure
HD: Hemodialysis
HIC: High income country
ICESR: International Covenant on Economic, Social and Cultural Rights
KRT: Kidney replacement therapy (dialysis and transplantation)
LIC: Low income country
LMIC: Lower middle income country
NCD: Non-communicable diseases
PD: Peritoneal dialysis
Pmp: Per million population
SDG: Sustainable Development Goals
SSA: Sub-Saharan Africa
UDHR: Universal Declaration of Human Rights
UHC: Universal health coverage
UMIC: Upper middle income country
WHO: World health organization
