# Supplementary material for: Challenges to the right to health in sub-Saharan Africa: reflections on inequities in access to dialysis for patients with end-stage kidney failure
Source: Int J Equity Health. 2022 Sep 5;21:126. doi: 10.1186/s12939-022-01715-3 (PMC9444088; doi:10.1186/s12939-022-01715-3)
Supplement: Supplementary file 1 — Additional file 1. [file 12939_2022_1715_MOESM1_ESM.docx]

**Supplemental File:**

**Purposive search terms utilized**

Many papers relevant for this review were identified through the prior Systematic Reviews on dialysis outcomes in Sub-Sharan Africa performed by 2 of the co-authors, GA and VL **(**reported in:  <https://www.thelancet.com/journals/langlo/article/PIIS2214-109X(17)30057-8/fulltext> and <https://www.thelancet.com/journals/langlo/article/PIIS2214-109X(15)00322-8/fulltext>)**.** Data bases searched included Pubmed and Web of Science, African Journals Online, WHO Global health Library.

For the additional purposive search the following terms were utilized in independent searches in Pubmed, Google Scholar, Google, qualified for Africa and sub-Sharan Africa:

human rights and kidney; human rights and dialysis; priority setting and kidney; priority setting and dialysis; rationing and kidney; rationing and dialysis; universal health coverage and kidney; universal health coverage and dialysis; economic burden and kidney; economic burden and dialysis; global burden of disease and kidney; moral distress and kidney; moral distress and dialysis; equity and kidney; equity and dialysis.

Additional references were identified through bibliography search and search for “similar articles” in Pubmed.

**Supplementary Table 1. Obligations and responsibilities of states and others***

| **Stakeholder** | **Obligation/responsibility** | **Relevance for dialysis** |
| --- | --- | --- |
| **State** | - Progressive realization | - Improve equity in access to dialysis - Health in all policies approach to optimize disease prevention and |
|  | - Respect | - Provide adequate public health measures to prevent CKD and AKI - Ensure access to early diagnosis and treatment to prevent ESKF - Transparent decision-making and communication |
|  | - Protect | - Minimize third party intervention, required integration within health system - Oversight of private dialysis |
|  | - Fulfill | - Legislation, financing, health system strengthening to prevent and treat kidney disease |
|  | - Accountability | - Transparent policy development and implementation - monitoring and evaluation of need for and quality of care - progressive expansion of services - combat corruption |
|  | - Monitor indicators: - Structural - Process - Outcome | - Transparent policy development regarding access to dialysis - Provision of infrastructure, training of primary care and dialysis staff - Access, mortality, renal registries |
| **Industry** | - Fairness | - Fair pricing of dialysis supplies - Reliable delivery - Refrain from incentives/manipulation |
| **Non-governmental organizations** | - Collaboration | - Avoid vertical dialysis programmes -> Integration within health system - Ensure long-term sustainability of dialysis is initiated |
| **International donors** | - Respect | - Do not “dump” used or useless dialysis machines - Ensure technical services in place - Ensure all components required for quality dialysis delivery are in place sustainably |
| **Society** | - Participation | - Hold governments accountable for omissions in prevention of kidney disease across spectrum of the SDGs - Advocacy - Be informed |
| **Nephrology work force** | - Participation | - Advocate for universal access to prevention and early treatment - Monitor and report in times fashion - Ensure maximal quality or care delivered - Informed consent |
| **Private sector** | - Participation | - Transparency - Fair pricing - Public-private partnerships for dialysis - Participate in reporting and tracking - Reinforce prevention messaging - Collaborate with public health sector |
| **Patients** | - Participation | - Advocacy - Be informed - Adhere to treatment - Ensure understanding |

*modified from UN/WHO Fact Sheets: **The Right to Health. Fact sheet No. 31.** [<https://www.who.int/gender-equity-rights/knowledge/right-to-health-factsheet/en/>]; **Human rights and health. Fact Sheet.** [<https://www.who.int/news-room/fact-sheets/detail/human-rights-and-health>]
